# Firm performance and cost of equity capital: the moderating role of narrative risk disclosure quality in Egypt

**DOI:** 10.1186/s43093-022-00156-2

**Published:** 2022-10-01

**Authors:** Tariq H. Ismail, Yousra R. Obiedallah

**Affiliations:** 1 Department of Accounting, Faculty of Commerce, Cairo University, Cairo, Egypt; 2 International Academy for Engineering and Media Science, 6th of October City, Egypt; 3 Department of Accounting, Faculty of Commerce, Sohag University, Sohag, Egypt

**Keywords:** Narrative risk disclosure quality, Cost of equity, Firm performance, EGX100, Egypt, C12, G12, G30, M41

## Abstract

This paper aims to examine the moderating effect of the narrative risk disclosure quality on the association between firm performance and the cost of equity capital in the Egyptian setting. Manual content analysis and factorial principal component techniques are used to quantify the quality dimensions of the narrative risk disclosures. The weighted average cost of equity is used to estimate the firms’ costs of equity. A cross-sectional analysis was conducted over three years (2018–2020) for a sample of 73 non-financial firms listed on the Egyptian Stock Exchange (EGX100). Multiple OLS regression models are employed to test the hypotheses. The results reveal a negative association between firm performance and the cost of equity and, while such association strengthened when adding the narrative risk disclosure quality as a moderator variable. This suggests that risk disclosure is important to stockholders’ investment decision-making in the Egyptian context. Based on the dearth of literature related to the economic reverberations of narrative risk disclosure quality in emerging economies, this study contributes to the risk reporting literature by providing evidence on the moderating effect of the narrative risk disclosure quality and its reverberations on the firm’s cost of equity capital in one of the emerging economies as Egypt. With regard to the findings of this study, we expect to contribute to the practice and theory by providing new and different insights about the moderating effect of narrative risk disclosure on the association between firm performance and cost of equity capital.

## Introduction

In the last two decades, regulatory bodies and professional associations have increasingly focused on the importance of narrative risk disclosure quality and its benefits to the firm’s internal and external users. Regulators and professionals believe that narrative disclosure quality is a needed step for enhancing firm credibility and accountability [11].

From a regulatory viewpoint, the US Security and Exchange Commission (SEC; [82, 83]) has forced all firms to incorporate a risk factors section in their annual filings (Sect. 1A of 10-K filings) to discuss *"the most significant factors that make the company speculative or risky."* The risk factor must be *"concise and organized logically"* to explain how the risk affects the firm’s securities being offered. Similarly, the Institute of Chartered Accountants in England and Wales (ICAEW; [48–50] has pointed out that investors need information about the firm risks to perform their own risk assessments and consider uncertainties when making their investment decisions. Similarly, firms would benefit from risk disclosure in their financing decisions.

Accordingly, research in developed countries has been motivated to investigate the economic reverberations of risk disclosure quality on a firm’s cost of capital and how investors perceive such disclosure in the capital market (e.g. [20, 43, 44, 76, 84]). In contrast, in the Egyptian setting, risk disclosure requirements are stated implicitly by some laws and some accounting standards, except for risk disclosures related to the firm's financial instruments (Capital Market Legalizations CML [29], Egyptian Institute of Directors EIoD, [30]; Egyptian Accounting Standards EAS: 40, [28]).

Thus, there is no specific risk disclosure standard in Egypt that clearly states the guidance for firms on how they should disclose different types of risks and related measurements in their annual reports. This, in turn, leads Egyptian firms to be reluctant to report all risk information in their annual reports due to the potential negative consequences.

Many theoretical and empirical studies have reported that overall disclosure quality reduces the costs of equity finance by lowering transaction costs or rising demand for the firm’s securities due to a reduction in information asymmetry between management and investors [16, 17, 26, 43]. Hence, risk disclosure quality is supposed to aid management in reducing the firm’s cost of finance and aid investors in their fund allocation (ICEAW, [48–50]).

However, the quality of narrative risk disclosure is a complex, multidimensional concept and immeasurable [81]. Prior studies followed one of two main methodologies to assess the quality of risk disclosure, which are subjective or objective measurements. Subjective measurements are based on questionnaires [61], or interviews [7], while the objective measurements are directed towards the source of the original information to obtain the required information, such as constructing an index based on the FASB and IASB [35, 36] qualitative characteristics of information [42, 80], or content analysis [11, 13, 33, 68].

In this regard, Botosan [18] stressed that the International Accounting Standards Board IASB’s qualitative characteristics provide good guidance on the assessment of information quality. However, the barrier to employing this measure is that one must assess each quality characteristic (relevance, reliability, understandability, and verifiability) from the perspective of each group of users involved in the risk disclosure, which will end up with excessive judgement and elevated costs. In contrast, Beattie [10] mentioned that using a composite quality measurement drawn from the IASB’s conceptual framework is retrograde and rigid, as it leads to multiple sub-concepts that are difficult to quantify.

It is worth noting that recent studies have mostly preferred to follow the content analysis approach (relying on multidimensional quality measurements) suggested by Beretta & Bozzolan [13] in assessing the reported risk information quality (e.g. [33, 68], Shivaani & Yadav, [86]) rather than self-constructed indices.

The main motivation for this paper arises from prior research calls for more studies to investigate the link between multidimensional risk disclosure quality and the firm’s cost of capital (Berreta & Bozzolan, 2004; [68]. Another motivation for this study is to examine the effect of the latest amendments to the Egyptian Accounting Standards (Decree No. 110/2015) and the Code of Corporate Governance [31] regarding risk disclosure requirements on the firms’ cost of equity. Thus, this study aims to answer the following question*: does narrative risk disclosure quality of the Egyptian firms’ annual reports moderate the association between firm performance and its cost of equity capital?*

This paper contributes to the existing risk disclosure literature in several ways. First, it responds to the calls of prior literature on risk reporting [13, 68] that recommended the adoption of multidimensional quality assessment of narrative risk disclosure and the extent to which it would affect the firm’s cost of capital in an emerging capital market. Second, this paper differs from prior research on risk disclosure in a number of ways. For Example, Rajab [77], Cabedo & Tirado [20], Hassan [42], Heinle & Smith [44], Sumardani & Handayani [87] examine the association between risk disclosure and cost of equity. Hence, this paper considers the impact of attributes of risk disclosure on firm performance. Additionally, Yuniarish & Triyonowati [90] assess and analyse the impact of corporate risk disclosure on cost of equity capital and to determine whether firm performance moderates the relationship between corporate risk disclosure and cost of equity capital. Unlike Yuniarish & Triyonowati’s [90], who do not examine the impact of multidimensional narrative risk disclosure quality on firm performance, this paper complements and extends such studies by looking at the moderating role of narrative risk disclosure quality on the association between firm performance and cost of equity capital in an emerging capital, Egypt.

The rest of this paper proceeds as follows: Sect. heading “Background” highlights background on the main concepts and revolution of risk reporting regulations in Egypt. Section heading “Literature review and hypotheses development” reviews the related prior literature and presents the development of the hypotheses. Section heading “Research design” illustrates the research design. Section heading“Empirical findings and discussion of results” discusses the empirical findings. Section heading“Robustness tests” presents the robustness tests. The final section contains the conclusions and suggestions for further research.

## Background

### Defining the cost of equity capital

The firm’s capital structure usually comprises of two vital external financing sources, equity (shares) and debt (loans and bonds) due to their ease of acquiring in the capital market (Nukala & Prasad Rao [72]). In Egypt, dealing with the cost of debt finance is obvious and easy to work out. Investors can adjust the interest rate of Treasury bills or loans for any tax benefits. However, the cost of equity financing requires accurate estimation to maintain the firm’s capital budget and decide whether a proposed investment will increase or reduce its stock price.

Botosan [19] defines the cost of equity as *"the minimum rate of return equity investors require for providing capital to the firm."* Heinle & Smith [44] defined it as *"the discount rate that is applied to prices relative to expected cash flows."*

### Defining risk disclosure quality

Sengupta [84] described risk disclosure quality as *"timely and detailed information that lowers lenders’ and underwriters’ perception of default risk."* While Beattie et al. [11] stated that the quality of risk disclosure is a complex and multisided concept, this complexity lies in the sensitive and subjective essence of the “quality” concept through the method by which disclosure is examined and assessed. Latterly, Rayan [79] defined risk disclosure quality as *“the provision of financial report information that better conveys the economic drivers (e.g. exposures to market risks, credit risks, liquidity risks, or information risks) and the statistical properties (e.g. covariance) of the variation in the firm's future economic performance."* This definition sheds light on the sources and the reverberations of the firm’s risk disclosure that could affect its future cash flows.

According to Botosan [18], the four requisite qualitative characteristics of financial information (relevance, reliability, understandability, and comparability) should be used in defining the “quality” of risk information due to their strong theoretical grounding in the FASB’s and IASB’s [51] conceptual frameworks. However, following this methodological approach is challenging and complicated in creating an empirical instrument to operationalize each of these quality characteristics. For example, *"quantifying the "reliability" of the firm’s disclosure through observing the economic substance of the transaction faithfully, as opposed to its legal form, free from bias, and complete within the bounds of materiality and cost, might be impossible in many settings"* [16].

Nevertheless, some prior studies relied on the IASB’s and FASB’s quality frameworks to assess firms’ risk disclosure quality (e.g. Oliveira et al., [42, 73]). Those studies operationalized some of the IASB’s and FASB’s quality characteristics, but not all of them. For instance, Hassan [42] neglects reliability in assessing the quality of Egyptian annual risk disclosures, while Oliveira et al. [73] found difficulty in assessing relevance, reliability, and understandability among the Portuguese credit institutions.

From another perspective, Beretta and Bozzolan [13] have pointed out four quality dimensions that could be used to define the "richness" of narrative risk disclosures. These dimensions are quantity, coverage, depth (qualitative–quantitative), and outlook. "Quantity" is the primary dimension of risk information quality, since many prior studies have found a positive association between the quantity and quality of risk information (e.g. [13, 61]). "Coverage" depicts the relative balance or concentration of risk disclosure across the different risk categories [11, 13, 68].

The two remaining quality dimensions, depth and outlook, signify the semantic properties of risk information quality. The "depth" dimension reflects qualitative and quantitative information about the expected economic effects of the firm’s reported risks on its future cash flows. "Outlook" reveals policies that have been taken or plans designed by management to mitigate the unfavourable consequences of a particular risk.

Beretta & Bozzolan's [13] proposed risk disclosure quality framework that was based mainly on the theoretical guidelines provided by (AICPA, [6], CICA, [21]; FASB, [34]; ICAEW, [49]) regarding voluntary risk disclosure. In addition, they considered academic recommendations provided by Robb et al. [78] and practitioner’s recommendations provided by Bell et al. [12] and Deloach [24] to strengthen their arguments regarding the suggested risk disclosure quality framework.

### Egyptian regulations arguing risk disclosure quality

In Egypt, several regulatory rules have been enacted to encourage new investments in the Egyptian capital market, revitalize the economy as a whole and strengthen investor confidence through enhanced transparency. For instance, the securities listing and de-listing rules of the Cairo and Alexandria Stock Exchanges (Decree of the Capital Market Authority’s Board of Directors No. 30—Dated June 18, 2002) stated that “*each company facing irregular material events that may affect its activity or financial position, and affect the trading of its shares at the Stock Exchange shall disclose these events immediately to the Stock Exchange within a specific time frame that allows the Stock Exchange to immediately publish such events on the brokers’ trading terminals”* (Article 24)*.* The article mentioned some examples of these "irregular material events," such as changes in the firm’s financing structure that involve an increase in the firm’s liabilities over its equity rights, or adding constraints imposed on the borrowing limit, changes in the firm's investment policies (including opening new branches, liquidating existing branches, shifting into leasing policy instead of owning some of the production tools), and lawsuits raised against the firm or any of its board members or directors.

Accordingly, firms should diversely disclose their potential risks into different categories that relate to the firm’s financial, operational, and strategic functions. This, in turn, supports the "coverage" and "depth" quality dimensions.

Similarly, the (EIoD—an affiliate of the Egyptian Financial Supervisory Authority (EFSA)—issued the Code of Corporate Governance (CG) in 2011 and updated it in 2016. According to rule No. 5-3-7 of the CG rules for private sector companies, the internal audit department is responsible for evaluating the methods and procedures for risk management. All risks (actual and potential) facing a company should be taken into consideration when creating its internal audit system and procedures. This is in line with the Code of CG for state-owned companies, which clearly states that companies should disclose information on risks and their impacts on the company’s economic and financial performance as well as risk management policies (rule 5–6).

In addition, the board of directors (BoD) should identify actual and potential risk strategy, how to deal with those risks, and the level of the firm’s risk appetite considering its size, its nature of activities, and the market in which it operates (rule No. 5-2-30 of CG). Consequently, CG rules aim to maintain internal risk management policies that enhance transparency regarding the firm’s potential risks and the planned strategies to face these risks, which, in turn, support the “outlook” quality dimension.

Regarding the newly adopted Egyptian Accounting Standards (EASs) that were issued by decree No. 110/2015 of the Minister of Investment to replace the previous accounting standards issued by decree No. 243/2006. The EASs have been prepared and issued in accordance with the International Financial Reporting Standards (IFRS), with some exceptions, where risk disclosure requirements can appear implicitly in some standards.

For example, according to item 125 of the (*EAS 1, Presentation of Financial Statements*), listed firms are required to disclose major assumptions used by the management that might impact firm's future performance and different sources of uncertainties linked to the management expectations at the end of each financial period that have a crucial impact on the next financial period. Furthermore, Article (129) of that standard stressed that these assumptions and potential risks should be reported in plain language to enhance understandability. Furthermore, firms should report the nature of these assumptions and risks, their sensitivity effects on other book values, their expected economic impact on the firm's assets and liabilities, and a description of any changes that occur to these assumptions in the event of the risk continuity.

Similarly, Article (21) of the *EAS 7 (Events after the Reporting Period*) states that listed firms are obliged to disclose information on the nature and financial impact of any event that occurs after the reporting period that might affect investors’ decisions. Article (22) of the standard stated some examples of these events, such as mergers and acquisitions of a big project or exemption of any branch, property damage resulting from fires, an announcement of a new restructuring, abnormal changes in the value of an asset or a liability, and lawsuits against the firm.

Article (8) of the (*EAS 15, Related-Party Disclosures*) mentions that firms should disclose information on the provisions for doubtful debts of the outstanding balances of related parties and any related risks that might affect the user’s assessment of the firm’s performance. Likewise, (*EAS 40, Financial Instruments: Disclosures*) addresses the disclosure of the risks related to the use of financial instruments. In addition, Article (32) states that firms should provide information on the nature, volume, and potential impacts of risks related to their financial instruments at the end of each reporting period. EAS (40) also states that firms should provide both quantitative and qualitative information relating to credit risk, liquidity risk, and market risk. This, in turn, supports the “depth” and “outlook” quality dimensions.

It is worth mentioning that the Egyptian CG rules and EASs are non-mandatory guidelines for firms planning to protect investors by balancing their interests against the interests of the firm’s management and enhancing transparency (EIoD, [30]). In addition, the EASs do not deal comprehensively with the requirements of firms’ risk disclosure; mainly, they lack clarity on the concept of risk, how different risks should be measured, and where they should appear in the annual report and classify different categories of risk.

## Literature review and hypotheses development

### The link between firm performance and cost of equity

Based on the prior literature, there are some points to clarify. First, there is a dearth of studies that explored the association between firm performance and the cost of equity capital. However, few studies, conducted in emerging countries explored the effect of the firm’s cost of equity on its performance. Second, all these studies document a negative association between the two variables.

Firm performance is considered a significant indicator of the firm’s financial health and its ability to obtain and allocate its resources to achieve a competitive advantage in the capital market [67]. In addition, it is vital information for internal and external users in investment decision-making. Prior studies used return on assets (ROA), return on equity (ROE), and Tobin’s Q to proxy firm performance [2, 43, 45, 52].

With regard to the cost of equity capital proxies, it is noted that prior research mostly followed two main approaches to calculate the cost of equity capital. The first approach is the CAPM (e.g. [2, 45, 55, 57, 89]), and the second approach is the dividend discounted cash flow or implied cost of equity capital. (e.g. [39, 23, 38], Easton [27]. However, in the Egyptian context, neither firms nor financial analysts released predictions for listed firms’ earnings per share or dividend growth rates, which are essential components to carry out any of these implied cost of equity models. Therefore, this study used the weighted average cost of equity capital (WACE) based on the CAPM due to its strong theoretical underpinnings.

Few prior studies examined the effect of the cost of equity on a firm’s performance (e.g. [2, 45, 52]). All these studies documented a negative association between cost of equity and firm performance. The results reveal that firm’s capital structure affects the firm performance.

However, only one study investigated the impact of performance on a firm’s cost of equity [89]. The results reveal no association between firm performance and cost of equity.

From agency theory and signalling theory perspectives, firm performance can reduce the information asymmetry between executives and investors, reducing investors' perceived risk and the firm’s cost of equity. Based on the above arguments, the first hypothesis can be formulated as follows:

#### H1

There is a negative association between firm performance and cost of equity in the Egyptian listed firms.

### Risk disclosure quality, cost of equity, and firm performance

The main theoretical underpinnings of the nexus between risk disclosure quality and the firm’s cost of equity capital are based on agency and signalling theories [62]. The two theories presume the existence of information asymmetry between executives and shareholders. Executive managers have an informational advantage over the shareholders, since they have access to the firm’s private information. According to Jensen & Meckling [53], the opportunistic behaviour of executives would cause a conflict of interests between owners and executives, thereby negatively affecting firms’ cost of capital and their riskiness. Thus, agency and signalling theories argue that the information gap between executives and shareholders can be reduced by more accurate disclosure [70]. Hence, revealing detailed and accurate risk information provided by management and how it alleviates these identified risks in the annual reports would reduce the information asymmetry between executives and shareholders [62].

Moreover, managers may need to signal their good performance through disclosing more accurate risk information and the mitigating polices taken to reduce potential losses in their reports presented to shareholders. Therefore, based on signalling theory, reported quality risk information increases the trust of executives' stewardship. In turn, this is expected to reduce cost of equity. Further, based on opportunistic hypothesis, executives may conceal their poor performance when the firm's risks are high and uncontrollable by decreasing the risk disclosure related to their reports. This, in turn, may have an impact on the cost of equity. Consistent with agency and signalling theories, the capital need theory also presumes that more disclosure can help in obtaining the required external finance at a lower cost [8, 15, 16, 22]. The logic behind this theory is the belief that a firm’s cost of capital can provide investors with some thoughts regarding the firm’s potential uncertainties through the currently reported information (FASB, [34]). Hence, the firm’s cost of capital decreases when shareholders can interpret the firm’s potential through more disclosure.

In this regard, the ICEAW [48] has mentioned that the need to report on risks and risk control measures can lead to improvement in accountability for stewardship and investor protection. This, in turn, will reduce information asymmetry between executives and shareholders and help firms obtain capital at lower costs. Consequently, risk disclosure quality is expected to have an impact on the association between cost of equity and firm performance.

A large body of accounting literature has concerned with the association between disclosure quality and the firm's cost of equity capital (e.g. [16, 37, 41, 43, 57, 60, 65, 75], [8]). However, these prior studies documented mixed results.

Similarly, few prior studies have examined the association between risk disclosure quality and the firm’s cost of equity and concluded with contradictory results. For example, in Italy, Cabedo & Tirado [20] found a negative association between the annual financial narrative risk disclosure and a firm’s cost of equity and no nexus between non-financial narrative risks and the cost of equity. This negative association between financial risk disclosure and a firm’s cost of equity is only significant when the date of the audit report is taken as a reference for the disclosure.

In the USA, Heinle & Smith [44] argued that risk disclosure quality can reduce investors’ perceptions regarding the firm’s future cash flows, which, in turn, leads to a reduction in the cost of equity capital. In the same vein, Sumardani & Handayani [87] and Yuniarish & Triyonowati [90] found a significant negative association between risk disclosures and Indonesian firm’s cost of equity capital over the period 2017–2019 for a selected sample of non-financial firms. Additionally, Yuniarish & Triyonowati’s [90] results reveal that firm performance has strengthened the interaction between risk disclosures and the cost of equity. This implies that more reported risk information by the firm can increase the equity market liquidity, which, in turn, will lower the cost of equity.

In contrast, Rajab [77] found no association between risk disclosure quantity and the UK firm’s cost of equity capital. Similarly, Hassan’s [42] results contradicted the theoretical arguments and revealed no association between the annual narrative risk disclosure and the Egyptian firm’s cost of equity.

In addition, little prior literature has examined the association between risk disclosure and firm performance, especially in emerging economies. These, previous studies concluded with conflicting results. For instance, Al-Dubai & Abdelhalim [5] examined the moderating effect of risk management disclosures on the association between the firm’s risk disclosures and its financial performance for 72 non-financial Saudi listed firms for the year 2018. The results indicated no association between risk disclosures and firm performance; however, after moderating the risk management disclosures, the results showed a significant positive association between risk disclosure and firm performance.

In contrast, some studies claimed that more reported negative risk disclosures in the firm annual reports will affect its financial performance severely [1, 9, 87].

Based on the above arguments, this study examines whether the relationship between firm performance and cost of equity is affected by the narratives risk disclosure quality as a moderator variable. Therefore, the second hypothesis is as follows:

#### H_2_

Narrative risk disclosure quality has a significant impact on the association between firm performance and cost of equity capital in the Egyptian listed firms.

## Research design

### Research method

The current study uses manual content analysis to assess the narrative risk disclosure quality of the Egyptian firms’ annual reports. This method is considered more accurate than automated content analysis in identifying certain features of specific information [46]. Content analysis methods include text, coding mode, coding unit, coding scheme, decision rules, and reliability and validity evidence [11]. In this study, the texts comprise the notes of the Egyptian firms’ annual reports. The coding mode employed is the manual one. "Sentence" is chosen as the unit of analysis, where it is considered the smallest integral unit of text that could convey an idea [13, 47, 59, 63].

Following the related risk disclosure prior literature, this study analysed sentences that contained "risk keywords" that communicate information concerning how any potential risks or prospects that have affected the firm's performance, or may affect it in the future are reported in the annual reports and any policies taken by the firm to alleviate these risks [3, 47, 59]. In addition, this study followed the ICEW [48] and Linsley & Shrives [63] risk categorization in the analysis as shown in Appendix 1. It is worth mentioning that Mokhtar & Mellett [69] used the same risk categorization in the Egyptian context. The coding scheme and decision rules are shown in Appendices (2) and (3), respectively.

This study used the ordinary least squares (OLS) regression, after controlling for both the year and industry fixed effects. In addition, the Durbin Watson statistics for Models (1) and (2) indicate no autocorrelation issues. Besides, the two models’ standard errors were heteroscedasticity-adjusted. Furthermore, all variables were winsorized at the 1% and 99% per cent levels to reduce the problem of outliers.

### Sample selection and data sources

The initial sample included firms that constitute the top 100 Egyptian firms in terms of liquidity and activity (EGX100). Banks and financial institutions (18 firms) were eliminated as they have different risk disclosure requirements and have specific financial characteristics. A firm to be included in the sample must meet the following criteria:

(i) It is listed on the EGX100 and has annual reports from 2018 to 2020 available on its website or at the Egypt Company for Information Dissemination (EGID), where this period witnessed the stability of Egyptian firms’ implementation of the Egyptian Accounting Standard amendments of the year 2015 and some amendments of the year 2019.
(ii) Has available financial data from Thomson and Reuters databases, specifically the weighted average cost of equity capital.

Not all the firms met such criteria; therefore, 9 firms have been omitted due to unavailability of their weighted average cost of equity capital in Thomson and Reuters or unavailability of their annual reports. After skipping those firms, the final sample size comprises (73) firms with 219 firm-year observations as shown in Table 1.

**Table 1:** Study sample

Description	Number of firms
Initial sample	100
Less: Financial firms	(18)
Non-financial firms	82
Less: Firms with missing financial data	(9)
***Final sample***	**73**

Annual reports of the selected sample were collected from firms’ websites and EGID for firms that did not publish the annual reports on the websites. Other financial data related to the firm’s cost of equity and other control variables were collected from the Thomson and Reuters database.

### Empirical models

#### Research variables

This study aims to examine the effect of firms’ narrative risk disclosure quality as a moderator variable on the association between firm performance and the cost of equity capital. Hence, four categories of variables are employed to achieve its objectives. The first category of variables is the dependent variable, which is the cost of equity capital (COE). The second category is the independent variable, which is the firm performance (ROA). The third category is the moderator variable, which is narrative risk disclosure quality. The fourth category of variables includes control variables, which include firm size, leverage, earnings quality, and liquidity. Table 2 presents the study variables and measurements.

**Table 2:** Variable definitions and measurements

Variable definition	Variable measurement
*Dependent variable*	
Cost of equity	Weighted average cost or equity capital based on the CAPM. It is calculated by multiplying the equity risk premium of the market with the beta of the firm’s stock, plus an inflation-adjusted risk-free rate. Hence, the equity risk premium is the expected market return minus the inflation-adjusted risk-free rate
*Independent variable*	
Firm performance	Return on assets (measured as: earnings before interest, taxes, depreciation, and amortization divided by total assets [43]
*Moderator variable*	
Risk disclosure quality	The principal component score of the highest eigenvalue computed from the factorial analysis of the principal components of the four quality dimensions
Quantity	Ln (total number of risk disclosure sentences)
Coverage	(1/ Herfindahl index) / the number of main risk categories
Depth qualitative	Ln (total number of qualitative risk disclosure sentences that have a predicted effect on the future cash flows of the firm)
Depth quantitative	Ln (total number of quantitative risk disclosure sentences that have a predicted effect on the future cash flows of the firm)
Outlook	Ln (total number of sentences include information about policies taken or planned strategies by the management to face the firm’s specified risk)
*Control variables*	
Size	Logarithm of firm market capitalization [16, 20, 43, 66]
Leverage	Long-term debt divided by the market value of common equity at the end of the year [56, 84]
Earnings quality	We used two measures to proxy firms’ earnings quality. First, the cross-sectional approach of the modified Jones model by [25], following Teoh et al. [88] steps to calculate current accruals quality. Second, the standard deviation of the firm’s earnings over 2017–2019 (Francis et al., 2008). Then, a principal component score with the highest eigenvalue calculated from two measures for earnings quality (firm’s accruals quality and rolling standard deviation of its earnings over the period from 2017–2019). Then, to adjust higher scores to represent higher earnings quality, the absolute values of the principal component scores are multiplied by (-1)
Liquidity	Quick ratio ((Total current assets – inventory) / Total current liabilities)

##### Measurement of cost of equity capital

To estimate the firms’ cost of equity, most prior studies in developed countries use the average of specific three or four different models of the implied cost of equity capital (e.g. [16, 20, 43, 75]). However, in the Egyptian context, neither firms nor financial analysts released predictions for listed firms’ earnings per share or dividend growth rates, which are essential components to carry out any of these implied cost of equity models such as Claus & Thomas [23], Gebhardt et al. [38], and Easton [27] models in the current study. Therefore, we used the weighted average cost of equity based on the CAPM framework due to its strong theoretical underpinnings. The CAPM explains how the firm risk is related to its expected returns [71]. Thus, the weighted average cost of equity is calculated by multiplying the equity risk premium of the market with the beta of the firm’s stock plus an inflation-adjusted risk-free rate. Hence, the equity risk premium is the expected market return minus the inflation-adjusted risk-free rate. This computation could be illustrated as follows [64, 85]:

$${R}_{i} ={r}_{f}+\upbeta ({R}_{m}-{r}_{f})$$

where ${(R}_{i})$is the expected return on stock (i), ${(r}_{f})$2.2is the risk-free rate, and $({R}_{m})$ is the risk premium or the excess rate of return above the riskless rate of interest (${r}_{f}$) on the stock (i). The β is the market risk, which represents the systematic risk inherent in the stock (i).

##### Measurement of firm performance and risk disclosure quality

We used the return on assets (ROA) to proxy the firm performance. ROA reflects the firm’s overall profitability; the most profitable firms are more likely to disclose more accurate risk information in their annual reports, thereby reducing information asymmetry and cost of equity. With regard to the risk disclosure quality, as discussed in Sect. heading "Defining risk disclosure quality”, prior studies have used various methodological methods to measure and assess the quality of risk information. However, we believe that using the multidimensional quality method in assessing the Egyptian firms’ annual narrative risk information could provide useful insights on shortcomings and suggestions that would improve the risk reporting in the Egyptian context.

Following [68], a factorial analysis of the principal components of the four quality dimensions is used to create a combined RDQ score. This combined RDQ score equals the principal component score of the highest eigenvalue. Our analysis showed only one factor with an eigenvalue higher than (1), it was factor1, which equalled (2.2596) eigenvalue and explains about 45.19% of the total observed variance.

However, there is a need to test the reliability and validity of the combined risk disclosure quality score. In two stages, we verified the reliability of our combined risk disclosure quality score. First, in line with Mokhtar & Mellet [69], we re-coded (30) randomly selected annual reports at a different time to test the coding stability. No changes were found in the coding classification scores. Second, we used Cronbach’s alpha as a statistical test to verify the reliability of the risk disclosure quality scores as used by Elshandidy & Shrives [32]. Cronbach's alpha is a measure that defines how a certain data set represents a unique internal consistency for a specific variable. The results revealed internal consistency between the combined RDQ score and the four quality dimensions, with a Cronbach’s alpha score of 78.77%. This score indicates that internal consistency is good since social sciences research generally accepts a rate of around 70%. [3].

To validate our risk disclosure quality measure, the coding scheme was executed based on detailed decision rules. These decision rules were adopted from prior research [3, 58, 63, 68], which are considered acceptable and accurate sources for the risk information classification among researchers.

##### Measurement of control variables

Based on the previous studies (e.g. [4, 16, 20, 40, 41, 65, 74]), we include four control variables. These variables are firm size (Size), idiosyncratic risk proxied by firm leverage (Lev), firm earnings quality (EQ), and firm liquidity (Liq). With regard to the firm earnings quality, we used two measures, the first measure is the cross-sectional approach of the modified Jones model by Dechow et al. [25] and Teoh et al. [88]. The second measure is the standard deviation of the firm’s earnings over 2017–2019 (Francis et al. [14]). All control variables’ measurements are given in Table 2.

#### Research models

This study aims to examine first the effect of firm performance on the cost of equity capital. Second, it investigates whether the risk disclosure quality has an impact on the association between firm performance and the cost of equity capital. Consequently, we run the following two main models. The first model is as follows:

1 $${\text{COE}}_{{{\text{it}}}} =\, \alpha_{0} + \beta_{1} ROA_{{{\text{it}}}} + \beta_{2} Size_{{{\text{it}}}} + \beta_{3} LEV_{it} + \beta_{4} EQ_{it} + \beta_{5} Liq_{it} + \varepsilon_{it}$$

where COE_it_ is the cost of equity of the firm (i), in the year (t),ROA_it_ is the firm (i) return on assets in the year (t),Size_it_ is the firm (i) size in the year (t),LEV_it_ is the firm (i) leverage in the year (t),EQ_it_ is the firm (i) composite earnings quality in the year (t).Liq_it_ is the firm (i) liquidity in the year (t),and Ɛ_it_ is the Random error.

To test the interaction effect of both firm performance and risk disclosure quality on the cost of equity capital, model (2) is used:

2 $${\text{COE}}_{{{\text{it}}}} =\, \alpha_{0} + \beta_{{1}} {\text{ROA}}_{{{\text{it}}}} + \beta_{{2}} {\text{RDQ}}_{{{\text{it}}}} + \beta_{{3}} \left( {{\text{ROA}}_{{{\text{it}}}} {\text{x RDQ}}_{{{\text{it}}}} } \right) + \beta_{{4}} {\text{Size}}_{{{\text{it}}}} + \beta_{{5}} {\text{LEV}}_{{{\text{it}}}} + \beta_{{6}} {\text{EQ}}_{{{\text{it}}}} + \beta_{{7}} {\text{Liq}}_{{{\text{it}}}} + \varepsilon_{{{\text{it}}}}$$

where RDQ_it_ is the principal component score with the highest eigenvalue for the risk disclosure quality indicators of firm (i) at time (t),

(ROA x RDQ) is the interaction between firm performance and risk disclosure quality (the moderator variable),

Other variables are as defined in the multiple regression Model (1).

## Empirical findings and discussion of results

### Descriptive and univariant analysis

Table 3 illustrates information about the descriptive statistics related to the study’s variables. As shown in Table 3, the results revealed a stability of the Egyptian listed firms’ costs of equity over the three years. On average, the firms’ cost of equity was 16% and 17% in the years 2018 and 2019, respectively. Then the average cost of equity moved down to 14% in the year 2020.

**Table 3:** Descriptive statistics of multiple regression model (1) and model (2) variables for years 2018, 2019, and 2020

Year / Variables	Mean			Median			Std. Deviation			Min.			Max.			Range		
	2018	2019	2020	2018	2019	2020	2018	2019	2020	2018	2019	2020	2018	2019	2020	2018	2019	2020
*Dependent variable*																		
Cost of equity	0.16	0.17	0.14	0.18	0.17	0.15	0.10	0.03	0.11	− 0.60	0.06	− 0.34	0.23	0.23	0.84	0.82	0.17	1.18
*Independent variable*																		
ROA	0.11	0.11	0.08	0.09	0.11	0.07	0.12	0.12	− 0.22	− 0.25	− 0.25	− 0.42	0.44	0.41	0.37	0.66	0.66	0.79
Quantity	4.26	4.24	4.30	4.26	4.28	4.33	0.74	0.61	0.61	1.95	2.08	2.08	7.60	5.39	5.35	5.66	3.31	3.27
Cover	0.40	0.40	0.42	0 .4	0.42	0.44	0.07	0.07	0.06	0.21	0.21	0.21	0.49	0.49	0.50	0.28	0.29	0.29
Depth Qual	0.14	0.14	0.60	0	0	0	0.42	0.42	0.87	0	0	0	2.20	2.08	2.48	2.19	2.08	2.49
Depth Quan	2.76	2.81	2.87	2.99	2.94	3.04	1.08	1.03	1.07	0	0	0	4.51	4.49	4.38	4.51	4.49	4.38
Outlook	2.01	2.02	2.09	2.08	2.08	2.08	0.61	0.67	0.63	0	0	0	3.33	3.13	3.26	3.33	3.13	3.26
RDQ Score	3.43	3.45	3.59	3.50	3.49	3.66	0.75	0.73	0.79	0.90	0.85	0.85	4.83	4.92	4.97	3.93	4.08	4.12
Size	14.4	14.24	14.2	14.3	14.1	14.1	1.76	1.83	1.67	10.39	10.12	10.80	17.29	17.81	17.36	6.89	7.69	6.56
Lev	0.16	0.20	0.25	0.01	0.03	0.05	0.41	0.43	0.51	0	0	0	2.56	2.67	3.16	2.56	2.66	3.16
EQ	− 0.08	− 0.09	− 0.06	− 0.05	− 0.06	− 0.04	0.08	0.10	0.05	− 0.48	− 0.56	− 0.22	− 0.001	− 0.001	− 0.001	0.48	0.56	0.22
Liq	2.02	1.88	1.90	0.96	0.96	0.93	5.95	6.14	6.17	0.05	0	0.11	50.75	53.18	53.20	50.70	53.18	53.09

The minimum values of the cost of equity of -60% and -34% for the years 2018 and 2020, respectively, imply that the lowest interest rates paid to investors were lower than the return by 60% in the year 2018 and by 34% in the year 2020. More precisely, a firm's negative cost of equity indicates that the firm has been negatively affected due to an increase in market inflation rates. It should be mentioned here that we followed prior research [42, 54] and excluded firms with a negative weighted average cost of equity capital (2 values only) from our final sample and reran Model 1, where no change in our results was found. Overall, the standard deviation values of the cost of equity were low, which indicates that the values of cost of equity tend to be close to the mean of the data set.

In addition, Table 3 presents the mean value of the firm’s financial performance as 11% in the years 2018 and 2019 and declined to 8% in the year 2020, with a minimum of  − 0.25, 0.25, and − 0.42, respectively, over the three years, and has a maximum of 0.44, 0.41, and 0.37, respectively, over the three years. This indicates that most of the Egyptian firms have lower profitability in the year 2020 due to starting of the COVID-19 pandemic.

With regard to the risk disclosure quality dimensions, the results of the descriptive statistics indicated higher mean and median values for quantity, depth quantitative, and outlook dimensions compared to the values for coverage and depth qualitative. This implies that Egyptian firms quantified the risk information to some extent and provided more details about the policies to face the identified risks. However, they were not concerned enough with providing full coverage for the different risk types or describing the potential economic impact on future performance.

In addition, the median values of the depth qualitative quality dimension revealed that about 50% of the firms did not provide a qualitative description of the expected economic impacts of the identified risks over the three years. With regard to the composite risk disclosure quality score, the highest values of the mean, median, standard deviation, and range are (3.59, 3.66, 0.79, and 4.12, respectively) for the year 2020. This indicates that the firms’ risk disclosure level was relatively high in the year 2020 compared to risk disclosures in the years 2018 and 2019. Appendix 4 provides examples of risk disclosure statements that are used in the narratives of annual reports of Egyptian-listed firms. Table 3, also, reports the descriptive statistics for the control variables.

Table 4 shows the correlation matrix between the tested variables. The cost of equity capital is not correlated with any of the independent, moderator, and control variables. All other independent variables’ variance inflation factor (VIF) values are less than 10, which indicate no multicollinearity problem among the independent variables in each of the cost of equity capital regression models. Furthermore, none of the correlation coefficients among the independent variables exceed 70%.

**Table 4:** Pearson correlation matrix

Variables	COE	ROA	RDQ	Size	Lev	EQ
ROA	− 0.094					
RDQ	− 0.035	0.114*				
Size	− 0.065	0.271***	0.394***			
Lev	0.044	− 0.217***	− 0.034	0.001		
EQ	− 0.097	− 0.099	0.058	− 0.021	0.147**	
Liq	0.02	− 0.037	− 0.438***	− 0.235***	− 0.077	− 0.065

No serious multicollinearity among the independent variables

*Significant at level 10%, **Significant at level 5%, ***Significant at level 1%

### Multivariate analysis

This section discusses the empirical results of the two multiple regression models that were formulated to test: (i) the association between the firm performance and cost of equity capital, and (ii) the impact of risk disclosure quality on the association between firm performance and the cost of equity capital. Table 5 reports the results of these two regression models.

**Table 5:** Multiple regression results

Variables	Model (1)		Model (2)	
	Coef.	*P*-value	Coef.	*P*-value
Constant	0.211	0.000***	0.182	0.006***
ROA	− 0.0605	0.060*	0.250	0.003**
RDQ	–	–	0.005	0.441
ROA x RDQ	–	–	− 0.101	0.002***
Size	− 0.002	0.542	− 0.001	0.632
Leverage	0.021	0.024**	0.022	0.029**
EQ	− 0.033	0.537	− 0.041	0.440
Liquidity	− 0.0003	0.147	− 0.0001	0.820
Year fixed effect	Included		Included	
Industry fixed Effect	Included		Included	
*R*^*2*^	7.58%		8.85%	
F-Values	5.14***		5.93***	
No. of Observations	219		219	

*Significant at level 10%, **Significant at level 5%, ***Significant at level 1%

The first model is significant at (*p* < 0.000); this signifies that the model explains the variation in the cost of equity. Model (1) *R*^*2*^ is 7.58% and Model (2) is 8.85%; this indicates a slight improvement due to adding the firms’ risk disclosure quality as a moderating variable in the model. The results of Model (1) reveal a significant negative association between firm financial performance and the cost of equity capital at a level of 10%. However, the coefficient of the firm performance (ROA) is weak ( − 0.0605). This implies that any change in the firm’s financial performance can affect its cost of equity capital. This result is consistent with the findings of [2, 45, 52], which indicate a negative association between the financial performance of the firm and its cost of equity capital. However, our results contradict Ur Rehman & Uz Zamn’s [89] results, which revealed no association between the two variables. Consequently, the first null hypothesis is rejected and Model’s (1) results support accepting *H*_*1*_.

In addition, our results indicate a significant positive association between leverage level and the firm’s cost of equity capital, which are consistent with those of Hail [41], Omran & Pointon [74], Lopes & De Alencar [65], and Cabedo & Tirado [20] and showed a significant positive association between the firm’s leverage level and its cost of equity at a level (*p* > 0.040). This can be explained on the ground that the higher level of leverage is perceived by investors as a higher level of risk, and hence, investors require a higher return. For other control variables, our results revealed no significant association between them and the firm’s cost of equity capital.

When considering the narrative risk disclosure quality as a moderating variable in the second model and testing its impact on the association between firm performance and the cost of equity, the model improved to some extent. Model’s (2) results reveal that the firm’s cost of equity reduces in cases in which firm financial performance is associated with high quality narrative risk disclosures. The coefficient of the moderator variable is ( − 0.101) and statistically significant at a level of 1%. Thus, this result supports the notion that in the Egyptian capital market the association between firm performance and cost of equity is affected by the quality of annual narrative risk disclosures.

This result is also consistent with the signalling theory assumptions, where disclosing high quality risk information which enhances transparent financial performance will lead to lower cost of equity. Consequently, Model’s (2) results support the acceptance of *H*_*2*_ and rejecting the second null hypothesis.

## Robustness tests

It is believed that further tests are required to eliminate the unobserved impact of endogeneity and heteroscedasticity in Models (1) and (2); hence, the static two-stage least squares regression 2SLS estimator is employed in this paper, where lagged firm size (t-1) and (t-2) are used as instrumental variables in our models. The results in Table 6 show similar conclusions of regression models presented in Table 5, where in Model (2), the results confirm that the interaction between firm performance and risk disclosure quality has a negative significant association with the firm’s cost of equity at a level of 10%. The empirical findings in Tables 6 reveal that accounting and market determinants of the firm performance associated with the cost of equity are the same regardless of the employment of 2SLS. Thus, these results reveal the absence of endogeneity problem and the absence of variables that could bias the relationship between the firm performance and the cost of equity in our sample firms.

**Table 6:** 2SLS Results for models (1) and (2)

Variables	Model (1)		Model (2)	
	Firm performance and cost of equity		Moderator RDQ	
	*Coef.*	*t*	*Coef.*	*t*
Constant	0.221	3.07***	0.193	2.55**
ROA	− 0.058	− 1.15	0.25	1.35
RDQ	–	–	0.006	0.60
ROA x RDQ	–	–	− 0.099	− 1.72*
Size	− 0.003	− 0.66	− 0.002	− 0.52
Leverage	0.021	1.19	0.022	1.23
EQ	− 0.033	− 0.46	− 0.041	− 0.56
Liquidity	− 0.0004	− 0.44	− 0.000	− 0.14
R-Squared	7.6%		8.8%	
No. of Obs	219		219	
Wu–Hausman F test	0.7822		0.7213	
Durbin–Wu–Hausman	0.7738		0.7093	
Sargan test Chi^2^ (1)	0.4606		0.5355	
Basmann test Chi^2^ (1)	0.4769		0.5525	

*Significant at level 10%, **Significant at level 5%, ***Significant at level 1%

Wu–Hausman and Durbin–Wu–Hausman are tests of endogeneity (the results reveal acceptance of H0: regressors are exogenous). Sargan and Basmann are tests of overidentification (the results indicate acceptance of the instrumental variables)

However, there is a need to test the timespan of the cost of equity; hence, we ran Models (1) and (2) for the cost of equity in the following year (COE _t+1_) while all other control variables remained at year (t). Thus, the cost of equity timespan changed from 2019 to 2021. Table 7 reports the results of this analysis. The interaction results in Model (2) confirming those reported in Table 5 of the regression models; however, the association between (COE _t+1_) and all control variables is significant except for earnings quality in Model (1) and for earnings quality and liquidity in Model (2).

**Table 7:** Regression models using cost of equity in the following year

Variables	Model (1)		Model (2)	
	*Coef.*	*P-value*	*Coef.*	*P-value*
Constant	0.138	0.000***	0.099	0.000***
ROA	− 0.065	0.039**	0.307	0.003***
RDQ	–	–	0.007	0.107
ROA x RDQ	–	–	− 0.122	0.002***
Size	0.003	0.017**	0.004	0.015**
Leverage	0.016	0.041**	0.017	0.039**
EQ	0.003	0.965	− 0.005	0.929
Liquidity	0.000	0.053*	0.000	0.813
Year fixed effect	Included	Included		
Industry fixed effect	Included	Included		
*R*^*2*^	10.6%	13.1%		
F − Values	8.47***	9.42***		
No. of observations	219	219		

*Significant at level 10%, **Significant at level 5%, ***Significant at level 1%

No serious multicollinearity in both models

Additionally, this paper conducted a robustness test based on a different proxy for the firm performance. We estimate our regressions using the Tobin’s *Q* as a proxy of the firm’s performance, where it is calculated as the accounting value of total liability plus stock capitalization to total assets [67]. Table 8 presents the results of testing the two research hypotheses. The results revealed some differences in Model (1), where it does not support H1. However, there is no difference in the results of Model (2), which confirm and support H2 as the narrative risk disclosure quality has a significant impact on the association between a firm’s financial performance and its cost of equity. Therefore, the robustness tests suggest that the proxies that are used to measure the firm performance would affect the relationship between the two variables; hence, firms should be careful in selecting the measure that expressing such variables.

**Table 8:** Robustness test of using Tobin’s *Q* to proxy the firm performance in the regression models

Variables	Model (1)		Model (2)	
	*Coef.*	*P-value*	*Coef.*	*P-value*
Constant	0.225	0.000	0.31	0.001
TQ	0.004	0.026	− 0.14	0.096*
RDQ	–	–	− 0.026	0.172
TQ x RDQ	–	–	0.044	0.089*
Size	− 0.003	0.333	− 0.003	0.264
Leverage	0.026	0.004	0.016	0.202
EQ	− 0.027	0.63	− 0.029	0.597
Liquidity	0.000	.145	− 0.001	0.253
Year fixed effect	Included		Included	
Industry fixed effect	Included		Included	
*R*^*2*^	7.3%		8.85%	
F-Values	4.78**		4.73***	
No. of observations	219		219	

*Significant at level 10%, **Significant at level 5%, ***Significant at level 1%

## Summary, conclusions, limitations, and suggestions for further research

Recently, research has been concerned with the economic reverberations of risk disclosure quality rather than its determinants. Meanwhile, prior research that has examined the effect of risk disclosure quality on a firm’s cost of equity by using a multidimensional quality measurement in Egypt is lacking. Hence, this study provides empirical evidence on the moderating role of narrative risk disclosure quality on the association between the firm financial performance and its cost of equity capital in the Egyptian context.

This study extends prior risk reporting literature calls [13, 68] by providing insights on the adoption of multidimensional quality of narrative risk disclosures and its effects on the firm’s cost of equity capital in an emerging capital market. This, in turn, would help to a better understanding of the importance of the narrative risk disclosures in the Egyptian capital market.

The results of the empirical study reveal a significant negative association between the firm’s financial performance and its cost of equity capital. This association between the two variables is strengthened when considering the narrative risk disclosure quality as a moderator variable. This suggests that reported risk information is essential for supporting investment decision-making in the Egyptian capital market.

Theoretical implications stem from the ongoing debate regarding the importance of narrative risk disclosures and the extent to which it would affect the firm’s cost of equity capital directly or indirectly. The findings of this paper also have some practical implications for regulators, managers, and investors. *First*, from the manual content analysis, the results revealed a low level of narrative risk disclosures in the Egyptian firms’ annual reports. More precisely, there was little coverage and qualitative descriptions about some types of the firms’ risks; therefore, there is a need to issue a new detailed risk disclosure standard that provides a clear framework and guidelines for risk disclosure to Egyptian firms. Hence, regulators in Egypt, such as the Ministry of Investment, should think about issuing a specific risk disclosure standard to provide recommended risk disclosure requirements as a simple, fast, and effective way to improve risk reporting of the Egyptian firms. *Second*, managers should improve their risk disclosure outlook and description and cover different types of risks in a specific section in the firm’s annual reports to improve the relevance of the reported risk information to stockholders and other users and lowering the firm’s cost of equity capital. *Third*, for expert investors (e.g. financial analysts or credit analysts), our findings provide some insights on how to assess the quality of the firm’s risk information.

This study has some limitations. *First*, we used the capital asset pricing model (CAPM) rather than other models of the implied cost of equity (e.g. Cluas & Thomas, 2001, Gebhardet et al., 2001; Easton [27]) in estimating the cost of equity capital. This was due to the unavailability of the estimated earnings per share or dividend growth rate, which are not provided by financial analysts operating in the capital market nor in the firm’s annual reports. It is worth mentioning here that Khlif et al. [57] also used the CAPM in estimating the cost of equity capital in the same context. *Second*, this study focuses only on analysing the quality of the firms’ annual reports narrative risk information and did not take into consideration other channels of risk disclosure. Hence, there is a need to carry out further research on the same theme using other risk disclosure sources such as the board of directors’ reports, financial releases, disclosure on social media, and online corporate governance reports. *Third*, this study did not focus on sub-classifications related to the tone and time orientation of the reported risk information. Thus, examining whether the tone of risk information would influence the investors’ reaction in the capital market is worth considering. *Fourth*, this study did not take into consideration the moderating effect of corporate governance attributes such as board structure, ownership structure, and audit committee or the propitiatory costs on the firm’s cost of equity. Thus, future research could also investigate the moderating effect of corporate governance structures on the association between the firm’s financial performance and its cost of equity capital. *Fifth,* the empirical analysis covers only three years from 2018 to 2020; therefore, there is a need for further research to carry out an event study to consider the time lag of COVID19 pandemic and its impact on the firm performance and the cost of equity in the Egyptian settings.

## Data Availability

Secondary sources of data are used to complete this study.

## Appendix 1: Risk disclosure coding scheme according to (ICAEW, 1998; [63]

**Table:**

**Financial risk**	Interest rate Exchange rate Commodity Liquidity Credit
**Operations risk** (arises from the firm’s structure, systems, people, and products or functions)	Customer satisfaction Product development Efficiency and performance Sourcing Stock obsolescence and shrinkage Product and service failure Environmental Health and safety Brand name erosion
Empowerment risk	Leadership and management Outsourcing Performance incentives Change readiness Communications
Information processing and technology risk	Integrity (e.g. the goodness of the firm information processing and technology risks) Access Availability Infrastructure
Integrity risk	Management and employee fraud Illegal acts Reputation
Strategic risk	Environmental scan (e.g. external environmental events that affect the firm’s environmental decisions. For example, new environmental laws) Industry Business portfolio (e.g. internal and external events affecting the firm’s portfolio decisions, such as merger and acquisition) Competitors Pricing (e.g. internal and external events that affect the firm’s pricing policy) Valuation Planning Life cycle Performance measurement Regulation (e.g. any changes in regulations that affect the firm’s decisions) Sovereign and political Compliance Litigation (e.g. lawsuits with the internal and external parties)

## Appendix 2: coding scheme of the risk disclosure quality dimensions

**Table:**

Risk categories/ quality dimensions	Financial risks	Non-financial risks					Total no. of risk sentences	Ln (scores)
		Operational risks	Empowerment risks	Processing and technology risks	Integrity risks	Strategic risks		
Quantity								
Coverage								
Depth qualitative								
Depth quantitative								
Outlook								

## Appendix 3: decision rules for risk disclosure coding scheme

The decision rules were adopted mainly from Abraham & Cox [3], Konishi & Ali [58], Linsley & Shrives [63], and Miihkinen [68] with slight changes to fit the Egyptian settings.

- To identify risk disclosure a modern definition of risk is to be adopted, which incorporates both the positive (gains) and negative (losses) outcomes of an event.
- Sentences are to be coded as risk disclosure if it contains any of the risk-related keyword, that depicts a firm’s crucial risk or opportunity, which has the potential to affect its future financial performance and its related mitigation strategies.
- A risk disclosure sentence is classified as:
  - *“Depth Quantitative”*: if it contains actual numbers, ratios, or percentages that imply an expected economic impact on the firm’s future performance.
  - *“Depth Qualitative”*: if it contains information that is not numerical in nature and implies an expected economic impact on the firm’s future performance.
  - *“Outlook”*: if it implies policies taken or planned strategies to face identified risk.

- Sentences are to be coded as risk disclosure if it shows present, past (backward-looking), or future (forward-looking) information.
- The risk disclosure shall be classified according to the coding scheme in Appendix (3), and by reference to Appendix (4) of risk categories.
- If a sentence has more than one possible classification, the information will be classified into the category that is most emphasized within the sentence.
- Tables (quantitative and qualitative) that provide risk information should be interpreted as one line equals one sentence and classified accordingly.
- Any disclosure that is repeated shall be recorded as a risk disclosure sentence each time it is discussed.
- If disclosure is too vague in its reference to risk, then it shall not be recorded as a risk disclosure.

## Appendix 4: Examples of risk disclosure in the egyptian firms’ narratives of annual reports

**Table:**

Firm Reuters code and annual report year	Risk disclosure sentence	Risk category	Semantic properties
CLHO (2019)	The management establishes a provision for ***impairment*** of 100% for default customers for more than 150 days from the claim date after deducting the amounts that expected to be collected after that date. It also creates a group-based provision based on historical ***failure*** rates	Operational risk	Outlook
ASEC (2019)	The ***potential effect*** of ***(Covid-19)*** on the firm performance is still ***uncertain***, however, for this year there is no ***significant effect*** on the firm outcomes	Strategic risk	Depth qualitative
TMG (2018)	The company manages liquidity ***risk*** by maintaining adequate reserves and borrowing facilities, by continuously monitoring forecasted and actual cash flows and matching the maturity profiles of financial assets and liabilities	Financial risk	Outlook
DSCW (2019)	The total amount of inventory ***impairment*** is ***7,649,960 EGP*** on 31 December 2019	Operational risk	Depth quantitative
SODIC (2018)	The company accepted to pay a total settlement amount of ***eight hundred million Egyptian pounds*** as a final and comprehensive settlement of all allegations raised ***against*** the company with respect to this issue The payment will be as follows: a.A payment of EGP 250 million upon signature of the settlement agreement b.Unequal four payments with a total of EGP 550 million, will be paid upon two years starting from 1, March 2019 and ended on December 1, 2020	Strategic risk	Depth quantitative
ARCC (2019)	At the end of each reporting period, the Group ***reviews*** the carrying amounts of its tangible and intangible assets to determine whether there are any ***indications*** that those assets have suffered an ***impairment loss***	Operational risk	Outlook
ETEL (2019)	The amount represented in the finance provided by Telecom Egypt to consortium Algerian de Telecommunication Company (CAT), where Telecom Egypt participates directly 50%. This company suffers from financial ***difficulties*** and sustains material ***losses***. The extraordinary General Assembly of (CAT) held on July 1, 2009, approved the ***dissolution*** and ***liquidation*** of (CAT). An ***impairment loss*** was formed for the full balance in the light of these circumstances since there is a high ***probability*** that Telecom Egypt ***will not be able to collect*** the finance given to consortium Algerian de Telecommunication company	Strategic risk	Depth qualitative
CLHO (2018)	During February and March 2018, the borrowing rate (corridor) ***decreased*** by 1% and 1%, respectively, which will ***affect*** the company’s liabilities regarding borrowings and finance interest	Financial risk	Depth qualitative
DSCW (2019)	Alexandria for Ready-made Garments (subsidiary company) has achieved a ***loss*** of ***3,093,666 EGP*** on December 31, 2019. As well as cumulative ***losses*** have exceeded half of the owners’ equity. This indicates a ***significant uncertainty*** about the firm’s going concern	Strategic risk	Depth quantitative and depth qualitative
EAST (2019)	The company has controlled smoke ***emissions*** by using cyclones with an impermeable flare to prevent ***emissions*** from dispreading in the air to protect the environment from ***pollution***, then reuse and recycle it by manufacturing naturalized smoke chips	Operational risk	Outlook

## Abbreviations

SEC: US security and exchange commission
ICAEW: The institute of chartered accountants in England and Wales
CML: Capital market legalizations
EIoD: Egyptian institute of directors
EAS: Egyptian accounting standards
IASB: The international accounting standards board
FASB: Financial accounting standards board
AICPA: The American institute of certified public accountants
EFSA: The Egyptian financial supervisory authority
CG: Corporate governance
BoD: Board of directors
ROA: Return on assets
ROE: Return on equity
WACC: Weighted average cost of equity capital
OLS: The ordinary least squares (OLS) regression
EGID: The Egypt company for information dissemination
COE: The cost of equity capital
RDQ: The risk disclosure quality
VIF: Variance inflation factor
2SLS: Two-stage least squares regression
